# The MoCA ‐ Language (MoCA‐L): A brief screening tool for language disorders in neurodegenerative diseases

**DOI:** 10.1002/alz70857_100236

**Published:** 2025-12-24

**Authors:** Monica Lavoie, Guillaume Duboisdindien, Joel Macoir, Ziad Nasreddine, Robert Laforce

**Affiliations:** ^1^ Research Chair on Primary Progressive Aphasia ‐ Fondation de la famille Lemaire, Quebec, QC, Canada; ^2^ Clinique Interdisciplinaire de mémoire, CHU de Québec ‐ Université Laval, Quebec City, QC, Canada; ^3^ Université de Franche‐Comté, INSERM, UMR 1322 LINC, Besançon, Franche‐Comté, France; ^4^ CERVO Brain Research Center, Quebec, QC, Canada; ^5^ Faculté de médecine, Université Laval, Quebec, QC, Canada; ^6^ MoCA Clinic and Institute, Greenfield Park, QC, Canada; ^7^ Clinique Interdisciplinaire de Mémoire, CHU de Québec‐Université Laval, Québec, QC, Canada

## Abstract

**Background:**

Commonly used screening tools such as the Mini‐Mental State Examination and the Montreal Cognitive Assessment (MoCA) examine global cognition but lack a specific focus on language functions. To address this gap, we developed the MoCA ‐ Language (MoCA‐L) to provide a brief screening tool for cognitive‐linguistic disorders such as primary progressive aphasia (PPA) and other neurodegenerative diseases.

**Method:**

A pilot study (Phase 1) was conducted with 15 healthy controls (7M/8F; mean age = 68.5 years old; mean education = 14.4 years) using a preliminary version of the test. This version comprised a comprehensive set of tasks and items designed to evaluate key speech and language functions functions in PPA and other neurodegenerative diseases (i.e. spontaneous speech, naming, semantics, repetition, sentence comprehension, syntax and grammar).

**Result:**

Mean and standard deviations were calculated for each item of every task. A consensus‐based process within our team of experts was then conducted. Items with higher difficulty (success rate < 90%) were eliminated. Then, the items included in the final version were chosen according to psycholinguistic parameters of interest. The test was narrowed down to a final version comprising six subtests: conversation (/3), picture naming (/8), semantic knowledge (/6), pseudowords and sentence repetition (/7), sentence comprehension (/4) and a picture description (/2), for a total of 30 points.

**Conclusion:**

The MoCA‐L, a brief 6‐subtests’ screening tool has been developed to improve the detection of language impairments associated with cognitive‐linguistic disorders such as PPA and other neurodegenerative diseases. In Phase 2, we plan to establish the psychometric qualities of the test, such as test‐retest fidelity, inter‐rater reliability, internal consistency and convergent validity. Phase 3 will focus on establishing normative data. Future plans also include the development of an interpretation guide as well as the adaptation of the MoCA‐L in various languages and cultures. The MoCA‐L will ultimately provide healthcare professionals with a simple and rapid screening tool designed specifically to assess language deficits in neurocognitive disorders, thus improving early diagnosis and management of these diseases.

